# Cosmetic outcome as rated by patients, doctors, nurses and BCCT.core software assessed over 5 years in a subset of patients in the TARGIT-A Trial

**DOI:** 10.1186/s13014-018-0998-x

**Published:** 2018-04-13

**Authors:** Tammy Corica, Anna K. Nowak, Christobel M. Saunders, Max K. Bulsara, Mandy Taylor, Norman R. Williams, Mohammed Keshtgar, David J. Joseph, Jayant S. Vaidya

**Affiliations:** 1 Radiation Oncology Clinical Trials and Research Unit, Comprehensive Cancer Centre, Sir Charles Gairdner Hospital, Nedlands, WA 6009 Australia; 2 Medical School, University of Western Australia, Nedlands, WA 6009 Australia; 3 Institute for Health Research, University of Notre Dame, 19 Mouat Street, Fremantle, WA 6160 Australia; 4 Radiation Oncology, Comprehensive Cancer Centre, Sir Charles Gairdner Hospital, Nedlands, WA 6009 Australia; 5 Surgical & Interventional Trials Unit, Division of Surgery & Interventional Science, Faculty of Medical Sciences, University College London, London, NW1 2FD UK; 6 Royal Free London Foundation NHS Trust, Division of Surgical Sciences, The Breast Unit, Pond Street, Hampstead, London, NW3 2QG UK; 7 Division of Surgery & Interventional Science, University College London, London W1W 7TS, UK; Whittington Hospital, Royal Free Hospital and University College Hospital, University College London, London, NW1 2FD UK

**Keywords:** BCCT.core, Breast Cancer, Cosmesis, Cosmetic Rating Systems, External Beam Radiotherapy, Targeted Intraoperative Radiotherapy, TARGIT-IORT

## Abstract

**Background:**

The purpose of this research was to assess agreement between four rating systems of cosmetic outcome measured in a subset of patients with early breast cancer participating in the randomised TARGIT-A trial. TARGIT-A compared risk-adapted single-dose intra-operative radiotherapy (TARGIT-IORT) to whole breast external beam radiotherapy (EBRT).

**Methods:**

Patients, their Radiation Oncologist and Research Nurse completed a subjective cosmetic assessment questionnaire before radiotherapy and annually thereafter for five years. Objective data previously calculated by the validated BCCT.core software which utilizes digital photographs to score symmetry, colour and scar was also used. Agreement was assessed by the Kappa statistic and longitudinal changes were assessed by generalized estimating equations.

**Results:**

Overall, an Excellent-Good (EG) cosmetic result was scored more often than a Fair-Poor (FP) result for both treatment groups across all time points, with patients who received TARGIT-IORT scoring EG more often than those who received EBRT however this was statistically significant at Year 5 only. There was modest agreement between the four rating systems with the highest Kappa score being moderate agreement which was between nurse and doctor scores at Year 1 with Kappa = 0.46 (*p* < 0.001), 95% CI (0.24, 0.68).

**Conclusion:**

Despite similar overall findings between treatment groups and rating systems, the inter-rater agreement was only modest. This suggests that the four rating systems utilized may not necessarily be used interchangeably and it is arguable that for an outcome such as cosmetic appearance, the patient’s point of view is the most important.

**Trial Registration:**

TARGIT-A ISRCTN34086741, Registered 21 July 2004, retrospectively registered.

**Electronic supplementary material:**

The online version of this article (10.1186/s13014-018-0998-x) contains supplementary material, which is available to authorized users.

## Background

Standard adjuvant treatment for women undergoing breast conserving surgery (BCS) is whole breast external beam radiotherapy (EBRT) delivered in 15–35 daily fractions over a period of 3–7 weeks [1–6]. Several countries have already adopted the use of Targeted Intra-Operative Radiotherapy utilising the Intrabeam device (TARGIT-IORT) as a form of partial breast irradiation for suitable women, which allows the delivery of radiation directly to the tissues at the site of the primary tumour in a single session at the time of wide local excision (WLE) or shortly afterwards. When compared to EBRT in the TARGIT-A Trial, TARGIT-IORT was found to be non-inferior in terms of local recurrence with no difference in breast-cancer survival and a small but significant improvement in non-breast-cancer survival favoring TARGIT-IORT. Unlike TARGIT-IORT delivered prepathology (during WLE), non-inferiority could not be established for postpathology TARGIT-IORT (separate to WLE), but the difference in local recurrence was not statistically significant [7]. Within a sub-group of the TARGIT-A trial (*n* = 342), cosmetic outcome based on objective measurements was found to be better with TARGIT IORT, particularly in the first year after surgery [8]. Previous analysis of the present dataset has shown similar cosmetic outcomes when comparing TARGIT-IORT to EBRT, but better breast-related quality of life, both as scored by patients [9].

Over time, a variety of methods to measure cosmetic outcome have been explored due to the importance of such secondary outcomes when assessing the acceptability of new treatments with similar efficacy to standard care. In 1979 Harris described a subjective assessment utilising a four point scale comparing the treated breast to the untreated breast. Objective measurements assessing breast retraction (BRA) were described by Pezner in 1985; then further developed by Christie in 2005 with the use of photographic assessment; then in 2007 by Fitzal with the Breast Analysing Tool (BAT) and by Cardoso with the development and validation of the BCCT.core software (Breast Cancer Conservative Treatment.Cosmetic results) [10–17]. Limited reproducibility of subjective results led to the investigation of objective measurements, however it has been argued that patient-assessed cosmetic outcome is the most important as it is the woman who must live with her cosmetic outcome, despite patients tending to score themselves more positively than their health care providers [11, 13, 18–20].

In the absence of a gold standard approach for assessing cosmesis, four existing and reasonably practical methods were utilised to compare cosmetic outcome between TARGIT-IORT and EBRT; a) subjective patient self-assessment, b) live subjective assessment by a nurse and c) a doctor (Radiation Oncologist), and d) digital photographic assessment to provide an objective measure of breast retraction, colour and scar; scoring for each assessment was based on the Harris scale [10]. This current report expands on previously reported subjective (patient self-assessment) [9] and objective (BCCT.core) outcomes [8] by focusing on agreement on cosmetic outcome between the different rating systems.

## Methods

### Patients and Treatment

As previously reported, 3451 patients from 33 centres in 11 countries participated in the TARGIT-A trial between 2000 and 2012 [7, 9]. Patients with early breast cancer suitable for breast conserving surgery were randomized to receive either a single dose of TARGIT-IORT (50 kV X-rays with INTRABEAM^(^™^)^ Carl Zeiss, Oberkochen Germany) or conventional 3–7 weeks’ EBRT. TARGIT-IORT patients with unfavourable pathology also received EBRT in ~ 15% of cases however these were excluded from this analysis. TARGIT-IORT dose to 1 cm was 5-6Gy (16-33Gy at applicator surface) and EBRT was conventional 3-dimensional conformal radiotherapy (45–50.4Gy in 15–28 fractions) [21].

This cosmesis sub-study includes 126 patients from 3 hospitals in Western Australia randomized predominantly in the postpathology setting. Relevant ethics approvals were obtained and all participants provided written informed consent.

Eligibility for Australian patients randomized postpathology was stricter than the main trial; unifocal invasive ductal < 2 cm tumours, node negative, hormone receptor positive, limited DCIS and no lymphovascular invasion. Fourteen EBRT and 4 IORT patients in this analysis were randomised before their WLE (prepathology stratification) where these stricter criteria did not apply hence some deviations are shown in Table 1.

**Table 1:** Baseline patient characteristics by treatment

*Patient, treatment and tumour characteristics*	TARGIT-IORT	EBRT
*Number of patients (% of total)*	60 (48%)	66 (52%)
*Age (mean years +/− SD)* *Range*	63 (+/− 8.2) 50–83	62 (+/− 7.4) 50–80
*Randomised prepathology (before WLE)* *Randomised postpathology (after WLE)*	4 (7%) 56 (93%)	14 (21%) 52 (79%)
*Baseline assessments prior to any surgery N (% of treatment group)*	2 (3%)	12 (18%)
*Baseline BMI (mean score kg/m* ^*2*^ *+/− SD)* *Baseline BMI Group* ^*a*^ *(% pf treatment group)*	29 (+/− 5.5)	30 (+/− 5.9)
*1 – Underweight (< 18.5)*	0%	0%
*2 – Normal (18.5–24.99)*	30%	16%
*3 – Overweight (25–29.99)*	30%	50%
*4 – Obese (30+)*	40%	34%
*Tumour Size (mm mean ± SD)* *Tumour Size Group, % of treatment group*	10 (+/− 4.2)	11 (+/− 5.0)
*< 11(mm)*	62%	52%
*11–20 (mm)*	38%	46%
*> 21(mm)* ^*b*^	–	1.5%
*Tumour Grade, n (% of treatment group)*		
*1*	37 (62%)	38 (57%)
*2*	23 (38%)	27 (41%)
*3*^*b*^	0	1 (1.5%)
*Tumour Type, n (% of treatment group)*		
*IDC* *Mixed IDC/ILC*^*b*^	59 (98%) 1 (1.7%)	64 (97%) 2 (3%)
*Lesions, n (% of treatment group)*		
*1*	60 (100%)	65 (98%)
*2*^*b*^	0	1 (1.5%)
*Extensive DCIS (> 25% of tumour + inside and out of tumour)* ^*b*^ *n (% of treatment group)*	0	4 (6.3%)
*Hormone receptor status, n (% of treatment group)*		
*ER + ve*	60 (100%)	64 (97%)
*PR + ve*	44 (73%)	52 (79%)
*ER and PR –ve* ^b^	0	2 (3%)
*Positive Nodes* ^*b*^ *, n (% of treatment group)*	0	1 (1.5%) (1 node)
*Largest Specimen Length (mean -mm +/− SD)* *Range*	89 (+/− 37.2) 25–205	89 (+/− 38.4) 40–267
*Extent of Axillary Surgery, n (% of treatment group)*		
*Nil*	3 (5%)	2 (3%)
*SLNBx*	49 (82%)	55 (83%)
*Clearance*	8 (13%)	9 (14%)
*Further Surgery Required, n (% of treatment group)*		
*SLNBx*	2 (3.3%)	2 (3%)
*Margins*	2 (3.3%)	7 (11%)
*Revision of Scar*	2 (3.3%)	0
*Radiotherapy Dose Range (Gy)*	16-33^c^	45–50.4
*Fractions (range)*	1	25 (25–28)
*Boost Given (20Gy in 10 fractions), n (% of treatment group)*	N/A	11 (17%)
*Supraclavicular Treatment, n (% of treatment group)*	N/A	1 (1.5%)
*Chemotherapy given* ^*b*^ *(n, % of treatment group)*	0	1 (1.5%)
*Baseline Cosmesis Scores (% Excellent-Good), mean (± SD)*		
Patient Harris	85 (+/− 0.36)	82 (+/− 0.39)
Nurse Harris	93 (+/− 0.25)	92 (+/− 0.27)
Doctor Harris†	87 (+/− 0.35)	100 (+/− 0.0)
BCCT.Core	83 (+/− 0.38)	90 (0.31)

Abbreviations: *WLE*: Wide Local Excision; *BMI*: body mass index; *DCIS*: ductal carcinoma in situ; *EBRT* = external beam radiation therapy; *ER*: estrogen receptor; *IDC*: invasive ductal carcinoma; *ILC*: invasive lobular carcinoma; *PR*: progesterone receptor; *SD*: standard deviation; *SLNBx*: sentinel lymph node biopsy; *TARGIT-IORT*: targeted intraoperative radiation therapy

^a^See reference 28; ^b^ Factors relevant only to the prepathology stratification; ^c^Dose to surface of applicator; † Significantly different (*p* = 0.003)

### Instruments and evaluations

#### Cosmesis Harris Scale

Patients completed a self-reported cosmetic assessment at baseline (before radiotherapy) and annually thereafter for five years as previously reported [9]. At the same time points, a Radiation Oncologist and a Research Nurse independently completed the same cosmetic assessment for each patient, blinded to other rater scores. Doctor and nurse raters were also involved in patient care and were not blinded to treatment allocation. At the same time-points, digital photographs were taken of the unclothed torso from neck to navel, frontal view, with the patient standing, a method used by others [12, 22]. All baseline measurements were taken before radiotherapy, the majority being after WLE, except 3% in the TARGIT-IORT group and 18% in the EBRT group that were taken before WLE (where patients were randomised before their WLE (prepathology) earlier in the study - prior to postpathology becoming the more common approach at the Australian study centres).

All cosmetic assessments used the Global Harris Scoring System (also known as the Harvard Scale) of Excellent, Good, Fair or Poor (Additional file 1: Table S1) [10, 18, 23, 24]. Responses were dichotomized into Excellent-Good (EG) or Fair-Poor (FP) categories in order to facilitate comparison with other published studies of cosmesis. The digital photographs were analyzed separately [8] utilizing the validated objective BCCT.core software [14–16, 25]. Scores are referred to in the subsequent text as doctor (Radiation Oncologist), nurse, patient and BCCT.core.

#### Analysis and Interpretation

Statistical significance was set at the level of *p* < 0.01 to account for multiple comparisons [26, 27].

IBM-SPSS-V22 (SPSS Inc.,Chicago, IL) was used for: non-parametric analysis (Mann-Whitney U-Tests and Chi^2^ tests) of raw unadjusted data, two sample t-tests for change between baseline and Year-5 scores, and inter-rater reliability analysis using the Kappa statistic to determine consistency among raters. Kappa scores were interpreted based on levels of agreement described by Landis and Koch; < 0 Poor, 0.0–0.20 Slight, 0.21–0.40 Fair, 0.41–0.60 Moderate, 0.61–0.80 Substantial, 0.81–1.00 Almost perfect [28]. Generalized estimating equations (GEE) with a variable covariance structure were used for the longitudinal dichotomized cosmesis endpoint scored by doctors and nurses using SAS-V9.3 (SAS Institute, Cary, NC).

## Results

**Fig. 1:**
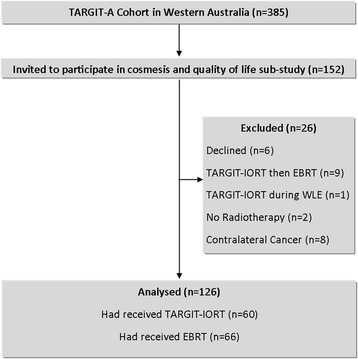
CONSORT diagram

Of the 385 Western Australian patients randomized into the TARGIT-A trial, the first 152 consecutive patients were invited to participate in this sub-study (further recruitment ceased due to resource constraints). Six declined participation; a further 20 were excluded due to confounders which would render cosmesis data uninterpretable, including (1) received both TARGIT-IORT and EBRT (*n* = 9); (2) received TARGIT-IORT during WLE (*n* = 1); (3) no radiotherapy given (*n* = 2); or (4) history of contralateral disease (*n* = 8). This left 126 evaluable participants, of whom 60 had TARGIT-IORT and 66 had EBRT (Fig. 1).

### Participants and Compliance

Initial compliance was very good and nearly identical across both treatment groups for all four rating systems but decreased over time. Availability of BCCT.core data at Years 3 and 4 fell below 50% and there was no data available at Year-5 (Additional file 1: Table S2).

Baseline patient characteristics were not different between treatment groups (Table 1).

### Cosmesis

At baseline, doctor scores for cosmesis were significantly better than results scored by patients in the EBRT group than the TARGIT-IORT group (100% vs. 87% Fisher’s Exact *p* = 0.003). Overall after treatment, a greater proportion of TARGIT-IORT patients scored an EG result compared to EBRT patients. Longitudinal multivariate analysis of cosmesis scores rated by doctors and nurses revealed no significant differences between patients treated with TARGIT-IORT and patients treated with EBRT (Additional file 1: Table S3). Un-dichotomized Harris Scale data are available in Additional file 1: Table S4.

Fisher’s exact Chi-squared univariate analysis for each rating system revealed three significantly different time points; patient Year-5 scores were the most divergent, with 90% and 68.4% scoring an EG response for the TARGIT-IORT and EBRT groups respectively (*p* = 0.042) [9], followed by the Year-2 Nurse scores (88.9% vs. 69.1%, *p* = 0.018) and then baseline doctor scores of 86.5% and 100% respectively (p = 0.003). Logistic regression with and without potential confounding variables (age, BMI, tumour size, tumour grade) did not alter these findings.

**Fig. 2:**
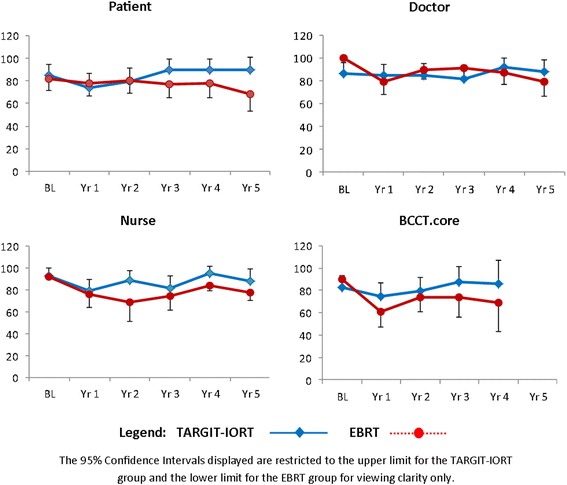
Cosmesis Outcomes (% Excellent-Good) by Rater. 95% Confidence Intervals displayed are the upper limits for the TARGIT-IORT group and the lower limits for the EBRT group

The proportion of EBRT patients achieving an EG outcome failed to return to the baseline proportion in the 4–5 years of follow-up across all rating systems however this was not statistically significant for any of the rating systems (Fig. 2). Patients who received EBRT also had a non-significant poorer outcome at Year-5 when compared to baseline for all subjective rating systems (*p* = 0.15 patients; *p* = 0.11 doctors; *p* = 0.05, nurses).

To test whether additional factors, including age, time since treatment, body mass index (BMI) and specimen size may have an impact on cosmetic outcome scores, Generalized Estimating Equation Models were constructed including these factors. Time since treatment and BMI were found to be related to cosmetic outcome at some, but not all time points (Additional file 1: Table S3) [29]. In the model examining nurse scores, cosmesis outcomes in Years 1, 2 and 3 were scored significantly worse than baseline (*p* = 0.004) for both treatment groups. Similarly, the model examining doctor scores found Year 1, 3 and 5 cosmetic outcome to be worse than baseline for both treatments (*p* = 0.018). Also in the doctor model, it was found that as patient BMI increased, the likelihood of scoring an EG result decreased (Estimate − 0.8, *p* = 0.009).

#### Review of agreement between cosmesis rating systems

Inter-rater reliability analysis revealed only 5 statistically significant Kappa scores out of the 36 observed time point pairings (Table 2). Four of these showed only fair agreement between raters. Only one time point scored moderate agreement which was between nurse and doctor scores at Year 1 with Kappa = 0.46 (*p* < 0.001), 95% CI (0.24, 0.68).

**Table 2:** Percentage agreement and Kappa scores for each rating system

Time-point	Patient vs. Doctor		Patient vs. Nurse		Patient vs. BCCT		Doctor vs. Nurse		Doctor vs. BCCT		BCCT vs. Nurse	
	% agree (n†)	Kappa (*p*-value)	% agree (n†)	Kappa (*p*-value)	% agree (n†)	Kappa (*p*-value)	% agree (n†)	Kappa (*p*-value)	% agree (n†)	Kappa (*p*-value)	% agree (n†)	Kappa (*p*-value)
BL	80.8 (99)	0.09 (0.59)	84.1 (107)	0.19 (0.05)	74.5 (98)	−0.07 (0.67)	88.3 (112)	0.07 (0.40)	85.1 (101)	0.15 (0.14)	81.8 (110)	− 0.01 (1.00)
Yr1	78.5 (93)	0.31 (0.006)**	78.8 (104)	0.37 (0.001)**	69.1 (97)	0.22 (0.034*)	83.3 (102)	0.46 (< 0.001)**	68.4 (95)	0.16 (0.15)	73.8 (107)	0.35 (< 0.001)**
Yr2	79.6 (98)	0.26 (0.018)*	70.8 (96)	0.12 (0.35)	63.3 (79)	−0.06 (0.75)	73.5 (102)	0.09 (0.46)	76.7 (86)	0.21 (0.048)*	75.3 (89)	0.31 (0.005)**
Yr3	73.2 (71)	−0.05 (1.00)	73.1 (78)	0.11 (0.45)	73.2 (41)	−0.01 (1.00)	73.8 (80)	0.17 (0.14)	80.8 (47)	0.22 (0.16)	76.0 (50)	0.35 (0.020)*
Yr4	81.1 (74)	0.20 (0.099)	78.9 (71)	0.01 (1.00)	66.7 (27)	−0.13 (0.60)	85.4 (82)	0.17 (0.17)	66.7 (27)	−0.13 (0.60)	72.4 (29)	0.25 (0.31)
Yr5	78.4 (51)	0.34 (0.027)*	75.0 (44)	0.27 (0.17)	–	–	80.4 (56)	0.24 (0.11)	–	–	–	–
Average Agreement	78.6		76.8		69.4		80.8		75.9		75.9	

**statistically significant at the *p* < 0.01 level; *statistically significant at the *p* < 0.05 level; †Number of scores available out of *n* = 126, not number of scores in agreement

**Fig. 3:**
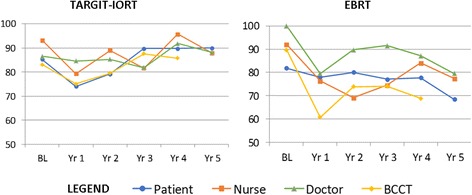
Cosmesis Outcomes (% Excellent-Good) by Treatment

One can see that most of the variation in responses was in the EBRT arm. BCCT.core correlated well with patient scores in the TARGIT-IORT group but not in the EBRT group (Fig. 3).

Figure 3 illustrates that each rating system followed a similar trend, with overall cosmesis scores showing 25% variation between raters within each of the 5 time points. Rater disagreement was seen such that doctors gave the most positive scores, followed by nurses, then patients and then BCCT.core. The significantly different time points (*p* < 0.01) between raters were Year 1 (doctors and BCCT.core both gave worse scores than nurses; doctors and nurses scored cosmesis better than patients) and Year 2 (BCCT.core gave worse scores than nurses) (Table 3).

**Table 3:** Inter-rater Reliability – Significant Kappa Scores

Time point	Rater Comparison (% EG)	Kappa	p-value
1 Year	Patient (76) vs. Doctor^ (82)	0.312**	0.006 ##
1 Year	Patient (76) vs. Nurse (78)^	0.366**	0.001 ##
1 Year	Patient (76)^ vs. BCCT (68)	0.222**	0.034 #
1 Year	Nurse (78) vs. Doctor (82)^	0.461***	< 0.001 ##
1 Year	Nurse (78)^ vs. BCCT (68)	0.345**	< 0.001 ##
2 Year	Patient (80) vs. Doctor (87)^	0.258**	0.018 #
2 Year	Doctor (87)^ vs. BCCT (77)	0.211**	0.048 #
2 Year	Nurse (79)^ vs. BCCT (77)	0.314**	0.005 ##
3 Year	Nurse (78) vs. BCCT (81)^	0.348**	0.020 #
5 Year	Patient (79) vs. Doctor (84)^	0.258**	0.027 #

**Fair agreement ***Moderate agreement ## *p* < 0.01 # < 0.05 ^scored higher proportion as EG

In terms of percentage agreement, nurse and doctor scores appeared to be the most closely related with an overall agreement of 80.8%; range 73.5% (Year 2) to 88.3% (baseline) (Table 2). The rating system that appeared most similar to the patient scores overall was the doctor scores, with an overall agreement of 78.6%; range 73.2% at Year 3 to 80.8% at baseline).

#### Sensitivity Analysis

The effect of missing data on the patient scores at Year-5 was tested by substituting the previous years’ result. For the EBRT group, this increased the proportion of an EG score from 68.4% to 69% and for the TARGIT-IORT group it decreased the proportion from 90% to 88%.

#### Discussion of results

Since its first use in 1998, intraoperative radiotherapy has been tested in randomised clinical trials and offered as adjuvant breast radiotherapy for over 20,000 women. Given the fact that breast cancer local recurrence outcomes are no different with TARGIT-IORT compared with EBRT, the obvious difference in patient experience (a single treatment instead of several weeks of daily treatments in the hospital) is of great importance.

Cosmetic outcome post various forms of intra-operative radiotherapy has been previously reported [8, 9, 30–34] however no study has compared four different rating systems, even with other approaches of breast conserving therapy. The earliest cosmetic assessment of TARGIT-IORT utilised a satisfaction index by asking patients to give a score for what she expected (E) and another for what she observed (O). It was found that for appearance, there was a trend for better scores for TARGIT IORT boost compared with EBRT and no difference between the two treatments was found for the satisfaction indices for texture [35, 36]. The current TARGIT-A sub-study assessed the agreement in cosmetic outcome between one objective and three subjective rating systems, by investigating the proportion of patients scoring an Excellent-Good (EG) outcome in each treatment group. Overall, the majority of patients in both treatment groups scored an EG cosmetic result across all scoring systems, however 32% (12/38) of EBRT patients assessed at Year-5 self-reported a Fair-Poor result. This compares poorly to the 90% of TARGIT-IORT patients (27/30 patients) self-reporting an EG result at this time-point, but nearly fits within the previously reported expectation that overall, 70–80% of EBRT patients will have an EG result [37]. Of those not providing a score at Year-5 due to having withdrawn (*n* = 3) or non-compliance (*n* = 11), 5 out of 7 from the EBRT group had a previous EG score and 3 out of 4 from the TARGIT-IORT group had a previous EG score, suggesting that had they provided a 5 year score which maintained their previous score, the proportions may have been 69% and 88% respectively. This suggests study attrition did not cause the large difference at this time point. Further discussion about the patient-reported findings has been detailed elsewhere [9].

It is well known that EBRT can have a negative impact on long term cosmetic outcome. This was confirmed in this study, with the EBRT group failing to reach baseline proportions of EG scores across all follow-up time points and all rating systems. Although follow-up scores when compared to baseline scores were not statistically significantly different between the TARGIT-IORT and EBRT groups in the subjective measurements, TARGIT-IORT patients did reach baseline proportions of EG scores in all rating systems, from Year-3 or 4 onwards. This suggests IORT patients do experience an initial decline in cosmesis but this improves beyond the second year of follow-up. Baseline assessments were taken prior to surgery in 1 patient in the EBRT arm (2%) and 12 patients (18%) in the TARGIT-IORT arm, which may introduce some bias in the interpretation of these results. Previously reported BCCT.core data showed patients receiving TARGIT-IORT were significantly more likely to have an EG result at Year 1 and Year 2, compared to patients who received EBRT [8]. The only time point to have moderate agreement between two raters was Year-1, between nurses and doctors, when cosmesis scores were poorer than at other time points. This suggests that for both treatment groups, cosmetic outcome assessed by all raters reached a nadir at Year-1.

Overall, a higher proportion of patients in the present study had an EG result compared to other different modalities of intraoperative radiotherapy. The Montpellier study (IORT delivered via a linear accelerator) reported ‘Excellent to Good’ cosmetic scores at 6 months and a Mammosite study (utilizing a balloon applicator) found 84% and 78% Excellent to Good scores at Years 1 and 2 respectively [30, 31]. Cosmetic assessments for these two studies were made by a clinician from physical assessments and photographic review and were not patient reported. Cosmetic outcome data from the ELIOT study (using 21Gy electron intra-operative radiotherapy) was reported as ‘good’ in the majority of cases, scored by both patients and clinicians [32]. A South African study delivering Iridium^192^ via after-loader found that 74% of patients reported an EG score after 7 years of follow-up [33].

Historically, patients have been known to evaluate cosmetic outcome more favourably than their clinicians, possibly due to a range of psychological factors; not wanting to displease their clinicians (or their teams) is a common suggestion. It may also be that factors other than aesthetics influence a patient’s evaluation of cosmetic outcomes; it may be related to quality of life, expectations or the difference in interpretation of what the Harris Scale means between different raters [20, 38, 39]. In this study, we found that patients’ self-assessments were similar to the objective assessment of the BCCT.core software, but only in those who received TARGIT-IORT, perhaps influenced by the better breast related quality of life with TARGIT-IORT [9]. Previous studies utilizing BCCT.core [17, 38, 39] have found that patients receiving EBRT score better than BCCT software. In this study, doctors were most likely to report an Excellent or Good outcome, followed by nurses. These results confirm previous research that subjective Harris Scale scores reported by the patient, her doctor and a nurse should not be used interchangeably [12, 13, 17].

Guidelines produced by the EORTC in 2006 stated that since there was no ‘gold standard’, at least 3 measures should be used to assess cosmetic outcome: a subjective panel of 5 members using the Harris Scale; some form of objective measurement system; and some form of skin damage grading, however this may not always be practical [17, 40].

Some authors have stated that, as patients have to live with the outcome of treatment, the patient self-assessments are the most important; although some contest that due to the low reproducibility of such results and the high dependence on psycho-social factors, they should be measured in conjunction with an objective measurement system (13, 17, 19, 34). An approach used in the past has been blinded review by two or more radiation oncologists, however the BCCT.core system was used instead of blinded review in the present study. Even though BCCT software is an objective assessment, we posit that the most practical and perhaps most relevant measurements are those carried out by the patient herself. This is particularly true when it is used within the context of a randomised trial, as all other factors would be equally balanced between the two arms and any effect would be attributable to the randomised allocation and should reflect the real-world scenario. It would be ideal to use all four methods in every study, or a combination of at least two, but as the other methods are more resource intensive and if they don’t correlate with the patient perception, they are arguably less relevant.

### Limitations and Strengths

During the design of the present study there was no standardised approach for measuring cosmesis post breast conserving surgery in randomised controlled trials [40]. At the time, a combination of several measures was considered better than one, hence four available and practical measures were undertaken (patient, doctor, nurse, and digital photographs in accordance with the Christie protocol [12, 22]). It was not until later that the BCCT.core software became available and subsequently applied to the photographs as a more contemporary computerised assessment technology than the originally planned Christie approach [8]. This study had excellent compliance rates for patient, nurse and doctor scores; however, digital photographs were not available for many patients towards the end of the study, which resulted in missing data for the objective cosmesis measurements. The impracticalities of annual photography contributed to the restriction of this sub-study to the first 152 patients registered in Western Australia [17] and reduced compliance in the later years, with the large geographical dispersion of patients in Western Australia potentially influencing return to the study centre. Image quality was also an issue, with some photographs not meeting the requirements for assessment by BCCT.core. The proportion of available BCCT.core data at Year 3 and Year 4 was only 44% and 27% respectively, hence the later BCCT results should be considered with caution. With current and future technology, and the awareness of the BCCT.core software, image quality should not be a problem for future prospective trials.

Doctor and nurse scores were not formally blinded to treatment received which may be a potential source of bias. Despite using a standard protocol, the doctor and nurse scores may also attract intra-rater bias as different doctors and nurses may have completed the cosmesis Harris scores. It was impractical to have the same assessors or photographers at each visit for the long duration of the study; however it was always the same patient assessing herself each time. This consistency is yet another argument to rely more on the patient’s own assessment than any other.

Another limitation is that there may be cultural differences in attitude to cosmetic outcome that may reduce the generalisability of the inter-rater results to different populations.

## Conclusion

As found in previous studies, a numerically higher proportion of patients treated with TARGIT-IORT had an Excellent-Good outcome compared with those who received EBRT. In this study, we found that there was little agreement between the four cosmetic rating systems used; in particular, patients’ score did not always correlate with the scores by doctors, nurses or the BCCT.core software. While on one hand, the objective assessment of cosmetic outcome used along with subjective assessments by staff and patients may be the ideal way to assess cosmesis, it can be argued that patient opinion of cosmetic outcome is the most important and may be the only outcome measured, particularly when resources are limited. In a randomised trial, the patient’s own assessment would give the most realistic measure of the difference in the cosmetic impacts of compared treatments.

## Additional file

Additional file 1: Supplementary Tables. (PDF 309 kb)

## Abbreviations

BAT: Breast Analysing Tool
BCCT.core: Breast Cancer Conservative Treatment, cosmetic results (software)
BRA: Breast Retraction Assessment
CI: Confidence Interval
EBRT: External Beam Radiotherapy (whole breast)
EG: Excellent-Good (cosmetic outcome)
FP: Fair-Poor (cosmetic outcome)
Gy: Gray (unit of measurement of radiation)
IBM-SPSS: Statistical Package for the Social Sciences (software)
IORT: Intraoperative RadioTherapy
ISRCTN: International Standard Randomised Controlled Trial Number
SAS: Statistical Analysis System (software)
TARGIT-A: Targeted intraoperative radiotherapy versus whole breast radiotherapy for breast cancer: an international randomised clinical trial
TARGIT-IORT: risk-adapted single-dose intra-operative radiotherapy utilizing the Intrabeam device
WLE: wide local excision
