# Supplementary material for: Cosmetic outcome as rated by patients, doctors, nurses and BCCT.core software assessed over 5 years in a subset of patients in the TARGIT-A Trial
Source: Radiat Oncol. 2018 Apr 13;13:68. doi: 10.1186/s13014-018-0998-x (PMC5899392; doi:10.1186/s13014-018-0998-x)
Supplement: Supplementary file 1 — Supplementary Tables. (PDF 309 kb) [file 13014_2018_998_MOESM1_ESM.pdf]

Additional File 1: Supplementary Tables

Table S1: Description of Harris Scale* Scoring System

**Score**	Description
**Excellent:**	Perfect symmetry, no visible distortion or skin changes<br><br>*(Treated breast appears almost identical to the untreated breast)*
**Good:**	Slight skin distortion, retraction or oedema; mild telangiectasia, mild hyperpigmentation, or an absent nipple-areolar complex<br><br>*(Minimal difference between the two breasts)*
**Fair:**	Moderate distortion of nipple or breast symmetry, moderate hyperpigmentation, prominent skin retraction, oedema or telangiectasia<br><br>*(Obvious difference between the two breasts)*
**Poor:**	Marked distortion, oedema or fibrosis, or severe hyperpigmentation<br><br>*(Marked changes in the treated breast)*

Also known as the Harvard Scale

Table S2: Cosmesis Compliance Tables by Time Point

Time	Treatment Group	Patients n	Patients %	Patients Chi^2 (exact)	Nurses n	Nurses %	Nurses Chi^2 (exact)	Doctors n	Doctors %	Doctors Chi^2 (exact)	BCCT.core n	BCCT.core %	BCCT.core Chi^2 (exact)
**BL**	IORT	54	90%	0.31	59	98%	0.62	52	87%	0.05	53	88%	1.00
	EBRT	55	83%		63	95%		64	97%		58	88%	
	Total	109	87%		122	97%		116	92%		111	88%	
**1Yr**	IORT	50	83%	0.44	58	97%	0.17	52	87%	0.48	52	87%	0.80
	EBRT	59	89%		59	89%		54	82%		56	85%	
	Total	109	87%		117	93%		106	84%		108	86%	
**2Yr**	IORT	48	80%	0.64	54	90%	0.43	54	90%	1.00	44	73%	0.84
	EBRT	55	85%		55	85%		58	89%		46	71%	
	Total	103	82%		109	87%		112	90%		90	72%	
**3Yr**	IORT	39	68%	0.14	49	86%	1.00	44	77%	0.82	24	42%	0.71
	EBRT	48	81%		51	86%		47	80%		27	46%	
	Total	87	75%		100	86%		91	78%		51	44%	
**4Yr**	IORT	39	70%	0.29	46	82%	0.64	49	88%	0.60	14	25%	0.83
	EBRT	45	79%		44	77%		47	82%		16	28%	
	Total	84	74%		90	80%		96	85%		30	27%	
**5Yr**	IORT	30	57%	0.23	33	62%	0.56	42	79%	0.38		N/A	
	EBRT	38	69%		31	56%		39	71%			N/A	
	Total	68	63%		60	56%		81	75%			N/A	
**Overall**	IORT	260	75%	0.05	299	86%	0.15	293	85%	0.84	187	64%	0.80
	EBRT	300	82%		303	82%		309	84%		203	65%	
	Total	560	78%		598	84%		602	84%		390	64%	

Abbreviations: BL = Baseline; IORT =TARGIT Intraoperative Radiotherapy group; EBRT = External Beam Radiotherapy group

Table S3: Generalized Estimating Equation Model Estimates, controlled for age, tumour size, tumour grade and BMI

Rater	Doctor			Nurse		
**Effect**	**Estimate**	**SE**	**p**	**Estimate**	**SE**	**p**
**Time Overall**	-	-	**0.018***	-	-	**0.004****
Year 1†	-2.1	0.7	0.003**	-1.4	0.5	0.006**
Year 2†	-1.3	0.7	0.049*	-1.4	0.5	0.003**
Year 3†	-1.5	0.6	0.013*	-1.4	0.6	0.009**
Year 4†	-0.7	0.7	0.304	-0.3	0.6	0.653
Year 5†	-1.6	0.7	0.018*	-0.9	0.6	0.112
BMI	-0.8	0.3	0.009**	-0.2	0.2	0.336
**Treatment**	0.7	0.5	**0.207**	-0.6	0.3	**0.083**

SE: Standard error BMI: Body Mass Index

† Compared to baseline result *significant at the 0.05 level ** significant at the 0.01 level

Bolded entries indicate overall GEE model p-values (Type III tests of fixed effects). Note that age, tumour size and tumour grade did not influence cosmetic result - estimates and p-values not shown

Table S4: Un-dichotomised Harris Scale Data

Time	Score	Patients IORT	Patients EBRT	Nurses IORT	Nurses EBRT	Doctors IORT	Doctors EBRT	BCCT.core IORT	BCCT.core EBRT
Baseline	Excellent	18%	27%	29%	46%	48%	55%	23%	24%
	Good	67%	55%	64%	46%	38%	45%	60%	66%
	Fair	13%	18%	5%	8%	14%	0%	17%	10%
	Poor	2%	0%	2%	0%	0%	0%	0%	0%
1Yr	Excellent	14%	17%	28%	24%	35%	32%	14%	5%
	Good	60%	61%	52%	52%	50%	48%	61%	55%
	Fair	24%	22%	21%	24%	15%	18%	25%	34%
	Poor	2%	0%	0%	0%	0%	2%	0%	5%
2Yr	Excellent	19%	24%	24%	27%	26%	31%	30%	15%
	Good	60%	56%	65%	42%	59%	59%	50%	59%
	Fair	19%	16%	11%	31%	15%	9%	20%	22%
	Poor	2%	4%	0%	0%	0%	2%	0%	4%
3Yr	Excellent	13%	23%	26%	31%	27%	19%	42%	22%
	Good	77%	54%	55%	43%	54%	72%	46%	52%
	Fair	10%	17%	18%	26%	18%	9%	12%	22%
	Poor	0%	6%	0%	0%	0%	0%	0%	4%
4Yr	Excellent	26%	18%	54%	46%	12%	26%	36%	25%
	Good	64%	60%	41%	39%	80%	62%	50%	44%
	Fair	10%	20%	4%	16%	6%	13%	14%	31%
	Poor	0%	2%	0%	0%	2%	0%	0%	0%
5Yr	Excellent	13%	26%	54%	42%	19%	20%	N/A	N/A
	Good	77%	42%	33%	36%	69%	59%	N/A	N/A
	Fair	7%	32%	12%	19%	10%	18%	N/A	N/A
	Poor	3%	0%	0%	3%	2%	3%	N/A	N/A
